# Severe Pneumatosis Intestinalis and Hepatic Portal Venous Gas in a Patient With Methamphetamine Use: Early Recognition and Management

**DOI:** 10.7759/cureus.68017

**Published:** 2024-08-28

**Authors:** Maria Theresa Lugue, Gabriel Cruz, Daniel T Jones, Manvir S Heer, Linsey Bui, Christopher Pace, Scott A Silver

**Affiliations:** 1 Internal Medicine, Touro University California, Vallejo, USA; 2 Internal Medicine, Touro University Nevada, Henderson, USA; 3 Internal Medicine, Kansas City University, Joplin, USA; 4 Internal Medicine, Valley Hospital Medical Center Internal Medicine Residency Program, Las Vegas, USA

**Keywords:** intensive care management, substance-induced complications, multisystem failure, critical care, radiographic imaging, metabolic acidosis, bowel ischemia, methamphetamine use, hepatic portal venous gas, pneumatosis intestinalis

## Abstract

Pneumatosis intestinalis (PI) and hepatic portal venous gas (HPVG) are rare but potentially life-threatening conditions characterized by the presence of gas within the bowel wall and portal venous system, respectively. This case report presents a 45-year-old male with a history of methamphetamine use who developed severe metabolic and hemodynamic instability, marked by altered mental status, metabolic acidosis, and ST elevations. Despite aggressive resuscitation and intensive care, the patient unfortunately succumbed to his condition, highlighting the gravity of these complications. This report underscores the importance of early recognition, comprehensive management, and timely surgical consultation to improve outcomes. It also emphasizes the need for a multidisciplinary approach and further research to better understand these conditions and the significant role of methamphetamine use as a contributing factor.

## Introduction

Pneumatosis intestinalis (PI) is an uncommon condition characterized by the presence of gas within the wall of the intestines, predominantly found in the small intestine and colon [1]. Hepatic portal venous gas (HPVG) is the presence of gas within the portal venous system, often indicating severe underlying pathology such as bowel ischemia [2]. PI can be categorized into primary (idiopathic) and secondary forms. Primary PI is rare, often asymptomatic, and generally benign [1]. Secondary PI is more prevalent and associated with conditions such as bowel ischemia, infections, inflammatory diseases, methamphetamine use, or trauma, necessitating prompt diagnosis and treatment. The pathophysiology of PI involves gas infiltrating the bowel wall through mechanisms such as increased intraluminal pressure, mucosal disruption, or bacterial production of gas [1]. HPVG results from mucosal disruption or bacterial translocation into the portal venous system, reflecting severe underlying pathology such as bowel ischemia [2].

Both PI and HPVG are uncommon but potentially severe findings. PI is detected in approximately 0.03% of adult patients undergoing abdominal radiography, more frequently identified through computed tomography (CT) imaging [1]. HPVG is rare, with an incidence of about 2.3% in patients with acute abdominal conditions requiring imaging [2]. Concurrent presentation of HPVG and PI is observed in less than 1% of patients undergoing abdominal imaging with CT scans, indicative of severe gastrointestinal conditions [3,4]. Concurrent presentation is also more common in elderly patients and those with significant comorbidities [3].

## Case presentation

A 45-year-old male was transported via emergency medical services (EMS) after being found with altered mental status (AMS) at a public location. Upon arrival, his vital signs included a temperature of 97.7°F, a respiratory rate of 28 breaths per minute, a blood pressure of 104/72 mmHg, an and oxygen saturation of 87% on room air. His heart rate was 200 beats per minute (bpm), and he was unable to answer questions appropriately. EMS had administered 12 mg of adenosine prior to arrival due to potential supraventricular tachycardia (SVT). The patient responded "yes" when asked about recent methamphetamine use. The patient met three out of four SIRS criteria (HR >90, RR >20, WBC >12,000/mm³). In the emergency department (ED), laboratory tests were conducted and reviewed showing metabolic acidosis (Table 1).

**Table 1:** Laboratory findings highlighting severe metabolic acidosis, electrolyte imbalances, and elevated tissue injury markers in a patient with pneumatosis intestinalis and hepatic portal venous gas WBC: white blood cell; ABG: arterial blood gas; CK: creatine kinase; Procal: procalcitonin; CRP: C-reactive protein

Test	Result	Units	Reference range
WBC	15,000	/µL	4,000-11,000
Lactic acid	36.0	mmol/L	0.5-2.2
Creatinine	6.19	mg/dL	0.6-1.2
ABG pH	7.33	-	7.35-7.45
ABG HCO3	18.4	mmol/L	22-26
ABG base excess	-7.3	mmol/L	-2 to +2
ABG pCO2	33.5	mmHg	35-45
Glucose	562	mg/dL	70-100 (fasting)
Sodium	150	mmol/L	135-145
Potassium	2.4	mmol/L	3.5-5.0
Chloride	84	mmol/L	98-106
CO2	16	mmol/L	22-29
CK	3,301	U/L	38-174
Procal	1.19	ng/mL	<0.1
CRP	1.8	mg/dL	<1.0

A positive urine drug screen confirmed methamphetamine use. An initial chest X-ray revealed no acute intrathoracic process, effectively ruling out conditions such as pneumothorax, pneumonia, pulmonary edema, pleural effusion, significant cardiomegaly, and any signs suggestive of aortic dissection. Despite receiving four liters of intravenous (IV) fluids, cefepime 1 g every 12 hours, and metronidazole 500 mg IV every eight hours per sepsis protocol, the patient began projectile vomiting dark brown emesis containing blood. His abdomen gradually became distended and tense, necessitating the placement of a nasogastric tube (NGT).

**Figure 1:**
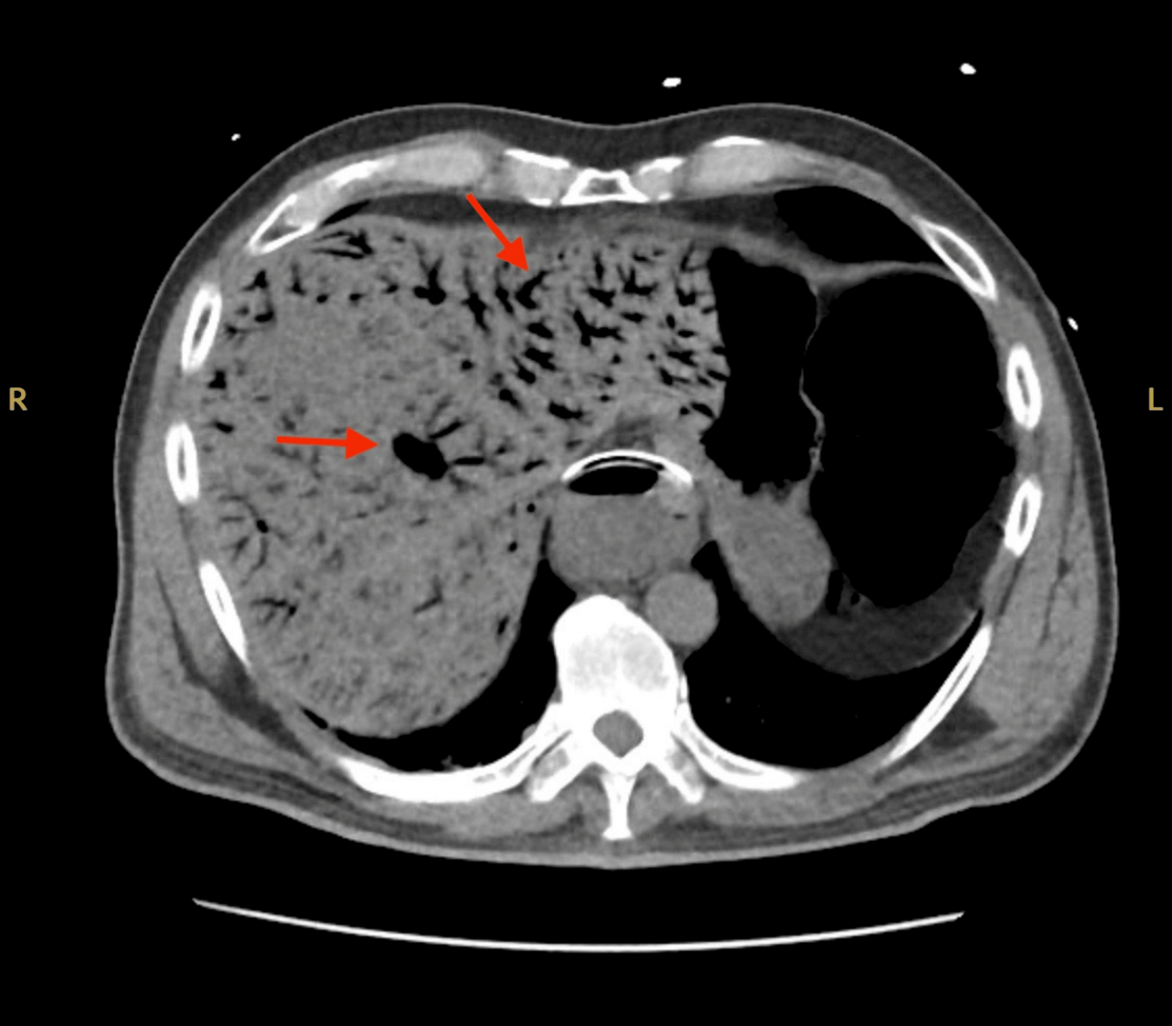
Axial CT scan of the abdomen and pelvis without contrast showing pneumatosis in the liver and portal venous gas

**Figure 2:**
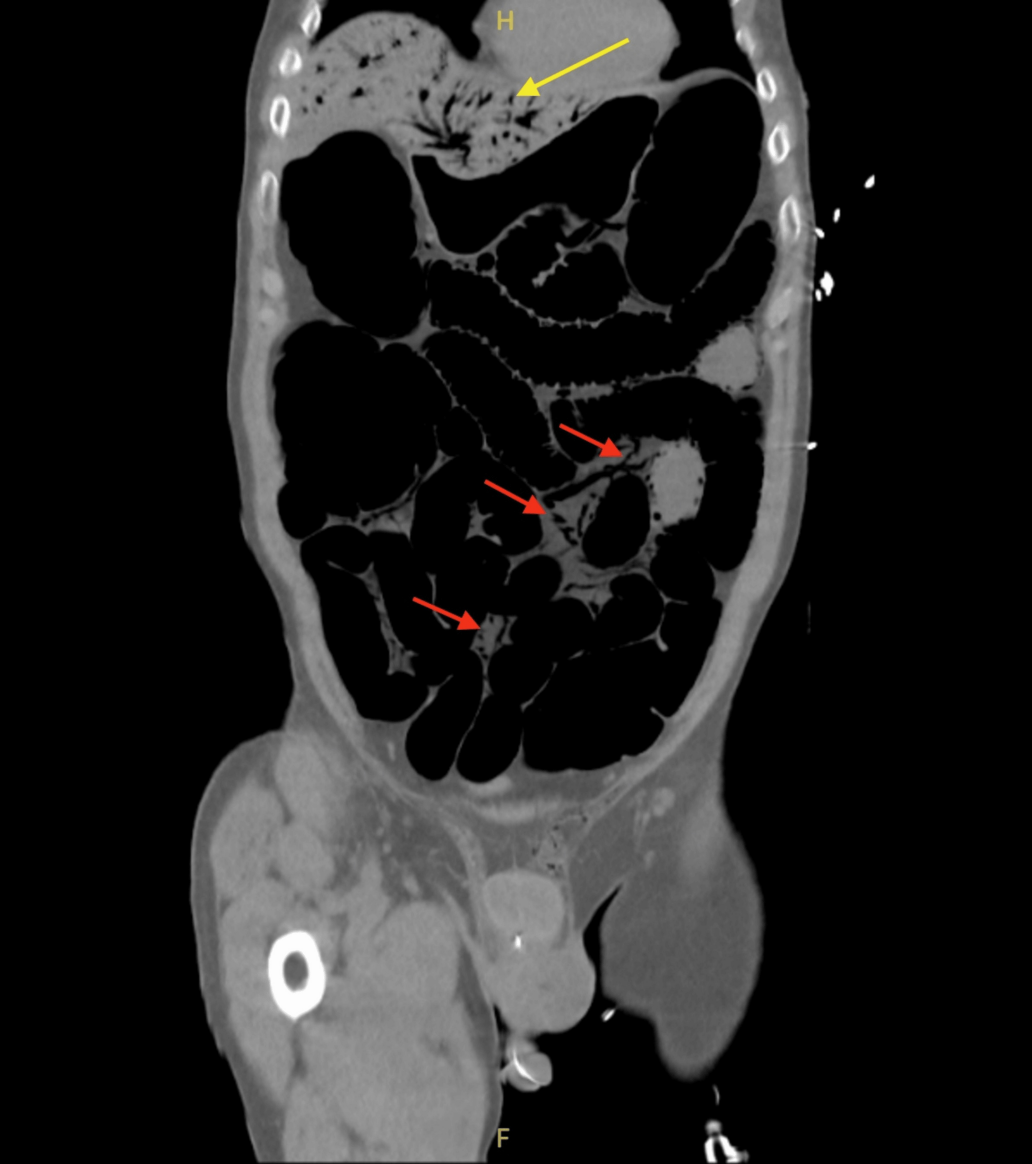
Coronal CT scan of the abdomen and pelvis without contrast demonstrating pneumatosis in the liver (yellow arrow), diffuse bowel distention, pneumatosis of the bowel (red arrows), and portal venous gas (yellow arrow)

**Figure 3:**
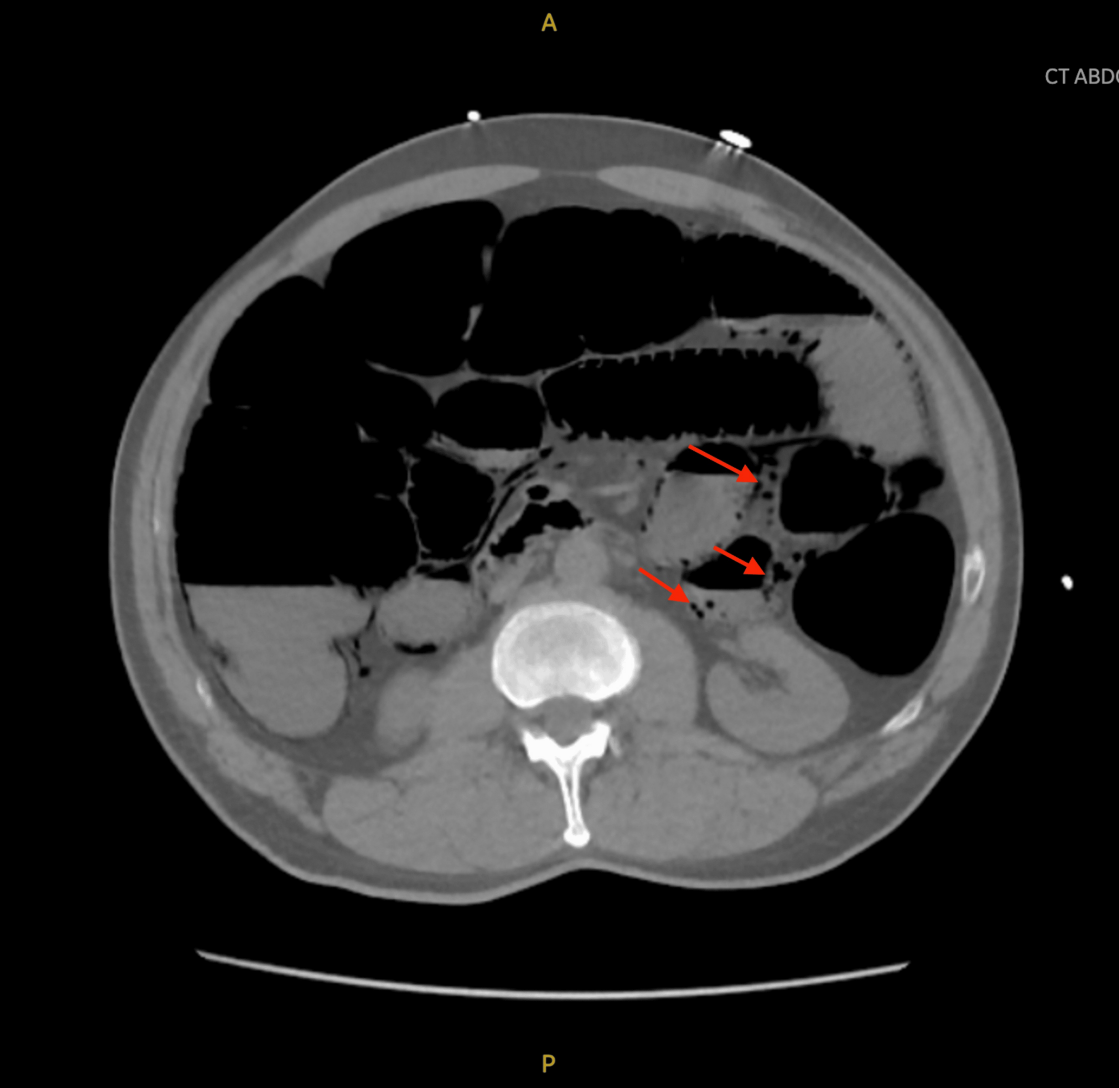
Axial CT scan of the abdomen without contrast demonstrating pneumatosis (red arrows) in the small bowel and diffuse bowel distention

A non-contrast CT scan of the abdomen and pelvis, performed due to concerns of abdominal distention and intestinal obstruction, revealed significant findings. The CT images demonstrated pneumatosis in the liver, diffuse colonic and small bowel distention with pneumatosis concerning for ileus, and portal venous gas (Figure 1, Figure 2, and Figure 3). The lower chest exhibited hypoventilatory changes suggesting reduced ventilation in certain areas of the lungs, but the liver, pancreas, spleen, adrenal glands, and kidneys appeared normal. There was no significant biliary ductal dilatation, and the aorta was nonaneurysmal. The CT scan revealed extensive distention of the bowel, along with the presence of pneumatosis and gas within the portal venous system. These radiologic findings suggest the presence of an ileus and raise the concern for a potential partial small bowel obstruction. While nonischemic causes could not be entirely excluded, ischemia was a concern. The general surgical team was consulted, but they determined that the patient was too unstable and would not survive surgery.

**Figure 4:**
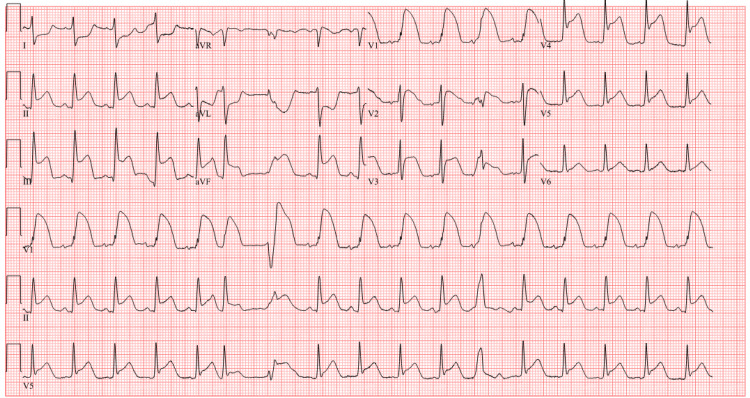
EKG showing sinus tachycardia with premature supraventricular complexes and occasional premature ventricular complexes EKG: electrocardiogram

Upon arrival in the intensive care unit (ICU), the patient became bradycardic, necessitating atropine administration, and a code blue was called. Return of spontaneous circulation (ROSC) was achieved after four cycles of compressions, approximately two minutes later. An arterial blood gas during the code revealed severe metabolic acidosis. An electrocardiogram (EKG) showed ST elevations in multiple leads, prompting a code ST-elevation myocardial infarction (STEMI) call, with a subsequent troponin level of 70.15 ng/L (Figure 4).

After the code STEMI was called, the cardiologist evaluated the patient, who had experienced bradycardia followed by unstable ventricular tachycardia (VT). This sequence likely contributed to the cardiac arrest, which was suspected to be secondary to the acute abdomen. Post-arrest, the patient exhibited ST elevations, prompting the activation of a code STEMI. However, a repeat EKG showed resolution of the ST segments, indicating that the initial elevations were likely due to demand ischemia in the context of the cardiac arrest. A stat echocardiogram showed a normal-sized left ventricle with normal global systolic function and an ejection fraction of 60-65% and noted left ventricular hypertrophy, which led to the deactivation of the STEMI alert. 

On ICU day 1, the patient was intubated and on continuous infusions of vasopressin and norepinephrine. Despite aggressive resuscitation efforts, the patient's condition remained critical, with ongoing concerns for ischemia and significant metabolic derangements. Further laboratory results indicated severe renal impairment and a rising lactic acidosis. Due to the patient's extreme instability, surgical intervention was deemed too high risk. Despite resuscitative efforts, the patient experienced a cardiac arrest on ICU day 2 and could not be revived.

## Discussion

This case presents a severe instance of concurrent PI and HPVG in a patient with a history of methamphetamine use. Several risk factors can predispose individuals to develop PI and HPVG, including bowel ischemia, severe gastrointestinal infections, inflammatory conditions like inflammatory bowel disease (IBD), traumatic or iatrogenic injuries, substance use (e.g., methamphetamines), chronic diseases like chronic obstructive pulmonary disease (COPD), and certain medications [1,2]. The rapid clinical deterioration, marked by metabolic acidosis, bradycardia, and ST elevations, underscores the critical nature of this presentation.

Typical symptoms of PI vary widely depending on the underlying cause. Patients may present with nonspecific gastrointestinal symptoms such as abdominal pain, bloating, diarrhea, and constipation. Severe cases may include gastrointestinal bleeding and signs of peritonitis [1]. Symptoms of HPVG are also variable and often related to the underlying condition. In the context of bowel ischemia, symptoms include acute abdominal pain, nausea, vomiting, and signs of sepsis [2].

Radiographic imaging, particularly CT scans, is crucial in diagnosing PI and HPVG. Key findings include linear or bubbly patterns of gas within the bowel wall for PI and branching radiolucencies within the liver for HPVG. Associated findings may include bowel wall thickening, mesenteric fat stranding, and signs of bowel ischemia [1,2]. Management involves addressing the underlying cause and providing supportive care. Aggressive resuscitation with IV fluids, medications to control heart rate and agitation, and supportive measures such as NGT placement and intubation were essential [3]. Given the patient's instability, surgical intervention was too risky, so intensive care focused on hemodynamic support and close monitoring [2].

Methamphetamine use can cause severe cardiovascular complications, including vasoconstriction, leading to mesenteric ischemia [4]. The hyperadrenergic state induced by methamphetamine use can exacerbate conditions like COPD and IBD, creating a scenario conducive to gas infiltration into the bowel wall and portal venous system [5]. The primary treatment for reperfusing ischemic bowel in this context involves the use of vasodilators, such as nitroglycerin or calcium channel blockers, to counteract the vasoconstriction and restore blood flow to the affected area. Other common causes of PI and HPVG include bowel ischemia, severe gastrointestinal infections, and inflammatory conditions [2].

A comprehensive literature review reveals that cases of PI and HPVG associated with substance use, particularly methamphetamines, are relatively rare but increasingly recognized. Studies document various complications of methamphetamine use, with mesenteric ischemia being a severe manifestation [3,6]. This case is unique in its presentation and the decision to forgo surgical intervention due to extreme instability, reflecting a tailored approach to the patient's condition. The management followed established clinical guidelines for the resuscitation and stabilization of critically ill patients, including aggressive fluid resuscitation and supportive measures like NGT placement and intubation [1,2]. Deviations from standard care, such as not performing immediate surgical intervention, were based on clinical judgment and the patient's instability.

Aggressive intervention is warranted in the presence of signs of bowel ischemia, severe metabolic acidosis, and hemodynamic instability. The decision to proceed with surgery must balance the risks of the procedure with the likelihood of survival. Immediate surgical consultation is necessary to determine the feasibility and potential benefit of operative intervention [1,2]. The mortality rate for patients with PI and HPVG secondary to bowel ischemia is high, often exceeding 50%, particularly when associated with severe metabolic derangements and multi-organ failure [2]. Mortality rates in cases of PI and HPVG vary widely, influenced by factors such as age, underlying conditions, and time to diagnosis. Early diagnosis and prompt intervention improve survival rates. Continuous advancements in imaging techniques and critical care management have improved recognition and treatment, potentially leading to better outcomes [1]. This case aligns with literature documenting the severe complications of substance use, including vascular and gastrointestinal effects [3,6]. It adds to limited reports of PI and HPVG in this context, highlighting the need for further research and awareness. The literature supports the importance of early diagnosis, aggressive management, and considering underlying causes like substance use.

## Conclusions

This case underscores the critical importance of early recognition and aggressive management of PI and HPVG, particularly in patients with potential contributing factors like substance use. The rapid progression to severe metabolic and hemodynamic instability highlights the need for timely intervention and the challenges in managing such high-risk cases. Early involvement of multidisciplinary teams and prompt decision-making regarding surgical intervention are crucial to improving patient outcomes. Continuous advancements in imaging techniques and critical care management have the potential to enhance early diagnosis and treatment, ultimately leading to better survival rates. This case adds to limited reports of PI and HPVG associated with substance use, emphasizing the necessity for further research and awareness in the medical community.
